# Successful treatment of metastatic bladder cancer by gemcitabine‐cisplatin re‐challenge after pembrolizumab

**DOI:** 10.1002/iju5.12348

**Published:** 2021-07-20

**Authors:** Takayuki Arai, Nobuyoshi Takeuchi, Tomokazu Sazuka, Hiroaki Sato, Yusuke Imamura, Shinichi Sakamoto, Akira Komiya, Tomohiko Ichikawa

**Affiliations:** ^1^ Department of Urology Chiba University Graduate School of Medicine Chiba Japan

**Keywords:** gemcitabine‐cisplatin re‐challenge, metastatic urothelial carcinoma, pembrolizumab

## Abstract

**Introduction:**

The advent of pembrolizumab has contributed to improved treatment outcomes for metastatic urothelial carcinoma, but the outcomes of treatments after second‐line treatment have not been established.

**Case presentation:**

A 72‐year‐old man was referred to our hospital with gross hematuria and diagnosed with suspicion of bladder cancer cT1N0M0. Transurethral resection of the bladder tumor was performed, but local recurrence and multiple lung metastases appeared 5 months after surgery. Although gemcitabine‐cisplatin was performed as first‐line chemotherapy, the local lesion increased, and pembrolizumab was used as a second‐line treatment. Pembrolizumab was also ineffective; however, re‐challenge with gemcitabine‐cisplatin as third‐line treatment produced a good therapeutic effect.

**Conclusion:**

We report a successful case in which gemcitabine‐cisplatin re‐challenge after pembrolizumab therapy was effective in metastatic bladder cancer. Re‐administration of chemotherapy after immune checkpoint inhibitors may be a broadly effective treatment option.

**Abbreviations**
CT: computed tomography
DCR: disease control rate
eGFR: estimated glemerular filtration rate
GC: gemcitabine‐cisplatin
ICIs: immune checkpoint inhibitors
MRI: magnetic resonance imaging
mUC: metastatic urothelial carcinoma
ORR: overall response rate
PD: progressive disease
PR: partial response
TURBT: transurethral resection of the bladder tumor

**Keynote message**

We report a case of metastatic bladder cancer that underwent gemcitabine‐cisplatin re‐challenge after pembrolizumab therapy. Re‐administration of gemcitabine‐cisplatin was effective and may be a treatment option.

## Introduction

In 2017, the use of pembrolizumab as a second‐line treatment for unresectable urothelial carcinoma was approved for insurance in Japan, expanding treatment options. However, its effect is limited and the prognosis for mUC remains very poor.^1^, ^2^ We report a case in which pembrolizumab was administered after GC to patients with advanced metastatic urothelial cancer, who were then re‐administered GC with a favorable therapeutic effect.

## Case presentation

**Fig. 1:**
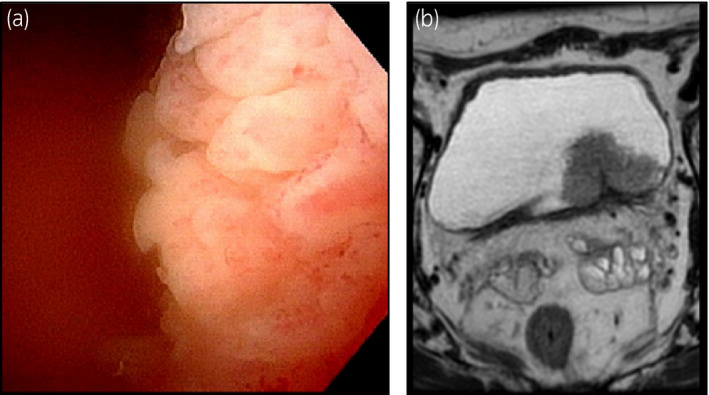
Image diagnosis at the first visit. (a) Cystoscopic findings: A 50‐mm papillary tumor extended from the bladder neck to the left wall. (b) MRI findings: A bladder tumor with no positive indication of muscular invasion was found on the left wall of the bladder.

A 72‐year‐old man visited a local doctor with a complaint of repeated gross hematuria and was referred to our hospital for suspicion of bladder cancer by ultrasonography. Cystoscopy revealed a 50 mm papillary tumor extending from the bladder neck to the left wall, and CT and MRI prompted a diagnosis of cT1N0M0 (Fig. 1). TURBT was performed, and the pathological diagnosis was invasive urothelial carcinoma, high grade (G2), and pT1. Following a second TUR, no residual tumor was found.

**Fig. 2:**
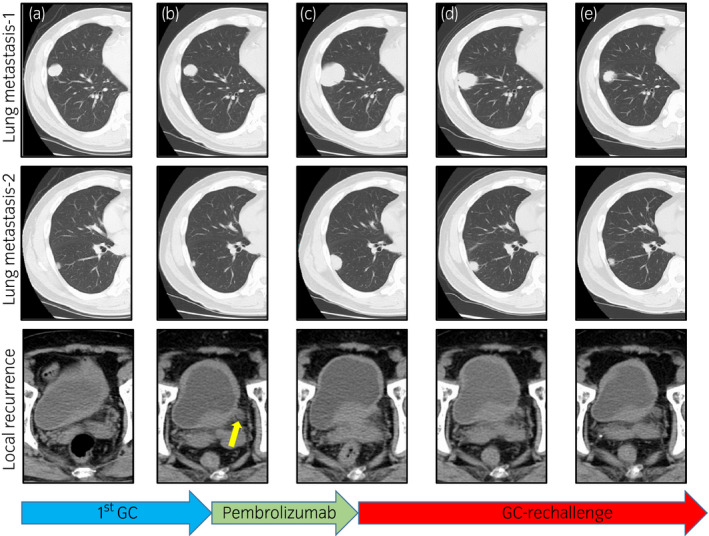
Treatment course after the appearance of lung metastases by CT evaluation. (a) Findings before the start of treatment. Multiple lung metastases and local recurrence are observed (eGFR = 75.7ml/min/1.73m^2^). (b) Findings after two courses of first GC. Multiple lung metastases did not change significantly, but local recurrence progressed. As shown by the yellow arrow, the left hydroureter was newly confirmed (eGFR = 65.4ml/min/1.73m^2^). (c) Findings after four courses of pembrolizumab. Multiple lung metastases were markedly increased (eGFR = 69.7ml/min/1.73m^2^). (d) Findings after one course of re‐challenge GC. Multiple lung metastases were significantly reduced, while local recurrence was unchanged (eGFR = 73.6ml/min/1.73m^2^). (e) Findings after three courses of re‐challenge GC. Multiple lung metastases were further reduced (eGFR = 73.3ml/min/1.73m^2^).

Bacillus Calmette‐Guérin bladder injection therapy for induction and maintenance was performed beginning at 1 month after the surgery, but recurrence in the bladder was nonetheless observed by cystoscopy 5 months after the surgery (Figure S1). In addition, bloody sputum appeared, and chest CT revealed multiple lung metastases (Fig. 2a). To treat the local recurrence of bladder cancer and multiple lung metastases, GC was started 6 months after the surgery. At the end of two courses of GC, the therapeutic effect was evaluated by CT; the lung metastases were almost unchanged, but a marked progression of bladder lesions was observed (Fig. 2b). The therapeutic outcome of GC was judged as PD. Two months after the start of GC, pembrolizumab was administered as a second‐line treatment. No obvious adverse events were observed during the period of pembrolizumab administration. Unfortunately, however, CT evaluation after repeating pembrolizumab administration every 3 weeks for four times showed a marked increase in lung metastases (Fig. 2c). Although we considered the possibility of pseudoprogression, we clinically determined it to be PD for the following reasons. First, chest X‐rays taken each time during pembrolizumab showed a clear increase in lung metastases over time (Figure S2), and finally it was evaluated by CT at a time more than 3 months after the start of pembrolizumab. In addition, exacerbation of bloody sputum symptoms was observed during the course. Therefore, deciding on chemotherapy and palliative medicine as the third‐line treatment, chemotherapy was selected because of the patient’s good general condition and strong desire for the treatment. We chose to re‐challenge with GC because there was no increase in lung metastases during first‐line GC treatment despite local progression; additionally, the results of other chemotherapy regimens at our hospital were not effective.

Evaluation by CT after 1 course of GC re‐challenge showed a reduction of lung metastases (Fig. 2d), so treatment was continued, and further reduction was confirmed at the end of three courses of GC re‐challenge (Fig. 2e). No local progression or appearance of new metastatic lesions was observed. The therapeutic effect is judged as PR, and GC re‐challenge is ongoing. During the first‐ and third‐line GC therapy, no major adverse events were observed, except for controllable myelosuppression and nausea.

## Discussion

The advent of the anti‐PD‐1 antibody pembrolizumab has expanded therapeutic strategies for metastatic urothelial carcinoma and contributed to improved prognosis. However, the therapeutic effect is limited, and there is no established treatment after this second‐line treatment.^3^, ^4^, ^5^ In this case, a metastatic bladder cancer became a progressive disease after GC therapy and pembrolizumab treatment, but re‐challenge with GC resulted in a partial response. Before the advent of ICIs, paclitaxel‐gemcitabine was often used as second‐line chemotherapy after GC therapy at our hospital. However, the response rate was poor at less than 10%, so re‐challenge with GC was performed as the third‐line treatment in this case.

Szabados *et al*. reported the response of chemotherapy after ICIs treatment in metastatic urothelial carcinoma. The maximum therapeutic effect was a 21% ORR and 82% DCR after performing chemotherapy as the third‐line treatment in patients with metastatic urothelial carcinoma who received sequence treatment of first‐line chemotherapy and subsequent second‐line ICIs. Platinum‐based chemotherapy was used for 100% of first‐line treatments and 79% of third‐line treatments.^4^

We believe that re‐challenge of GC, as in our case, may have a therapeutic benefit. We inferred three possible reasons why GC re‐challenge was effective in this case. The first is that GC was made effective by prior administration of pembrolizumab, the second is that first‐line GC was actually partially effective, the third is that the effect of pembrolizumab may have been delayed. From the course of treatment, although there was an increase in local lesions at first GC, lung metastases were unchanged, so it is entirely probable that there was a partial therapeutic effect. However, the marked reduction of lung metastases with GC as a result of re‐challenge suggests that pembrolizumab administration may have had a better therapeutic effect. Previous reports have also shown that ICIs treatment alters the tumor microenvironment, thereby enhancing the therapeutic effect of subsequent chemotherapy.^6^, ^7^ The mechanism is that immunotherapy, which removes the inhibition exerted by tumor cells or other immune cells, leads to increased accumulation of helper T cells at the tumor site and maintains an immune response. As a result, it is speculated that the killing of tumor cells via subsequent cytotoxic chemotherapy is enhanced.^8^, ^9^, ^10^ Dwary *et al*. also reported that there are many cases in which a favorable response was obtained with third‐line chemotherapy following first‐line chemotherapy and second‐line ICIs.^7^

## Conclusion

We report a successful case in which GC re‐challenge after pembrolizumab therapy was effective in metastatic bladder cancer. As in this case, GC re‐challenge after ICIs treatment can be one of the treatment options; however, further verification is needed in the future.

## Conflict of interest

The authors declare no conflict of interest.

## Approval of the research protocol by an institutional review board

The protocol for this research project has been approved by a suitably constituted Ethics Committee of the institution, and it conforms to the provisions of the Declaration of Helsinki.

## Informed consent

Informed consent for publication was obtained from the patients.

## Registry and the registration no. of the study/trial

This case report was approved by the Institutional Review Board of Chiba University Hospital (IRB No. 2554).

## Supporting information


**Figure S1**. Cystoscopic findings at the time of recurrence. Non‐papillary elevated lesions were found on the left wall of the bladder.

**Figure S2**. Treatment course for lung metastases during pembrolizumab. Chest X‐rays taken each time during pembrolizumab showed a clear increase in lung metastases over time.
