# Supplementary figures and images for: Successful treatment of metastatic bladder cancer by gemcitabine‐cisplatin re‐challenge after pembrolizumab
Source: IJU Case Rep. 2021 Jul 20;4(6):360–2. doi: 10.1002/iju5.12348 (PMC8560445; doi:10.1002/iju5.12348)

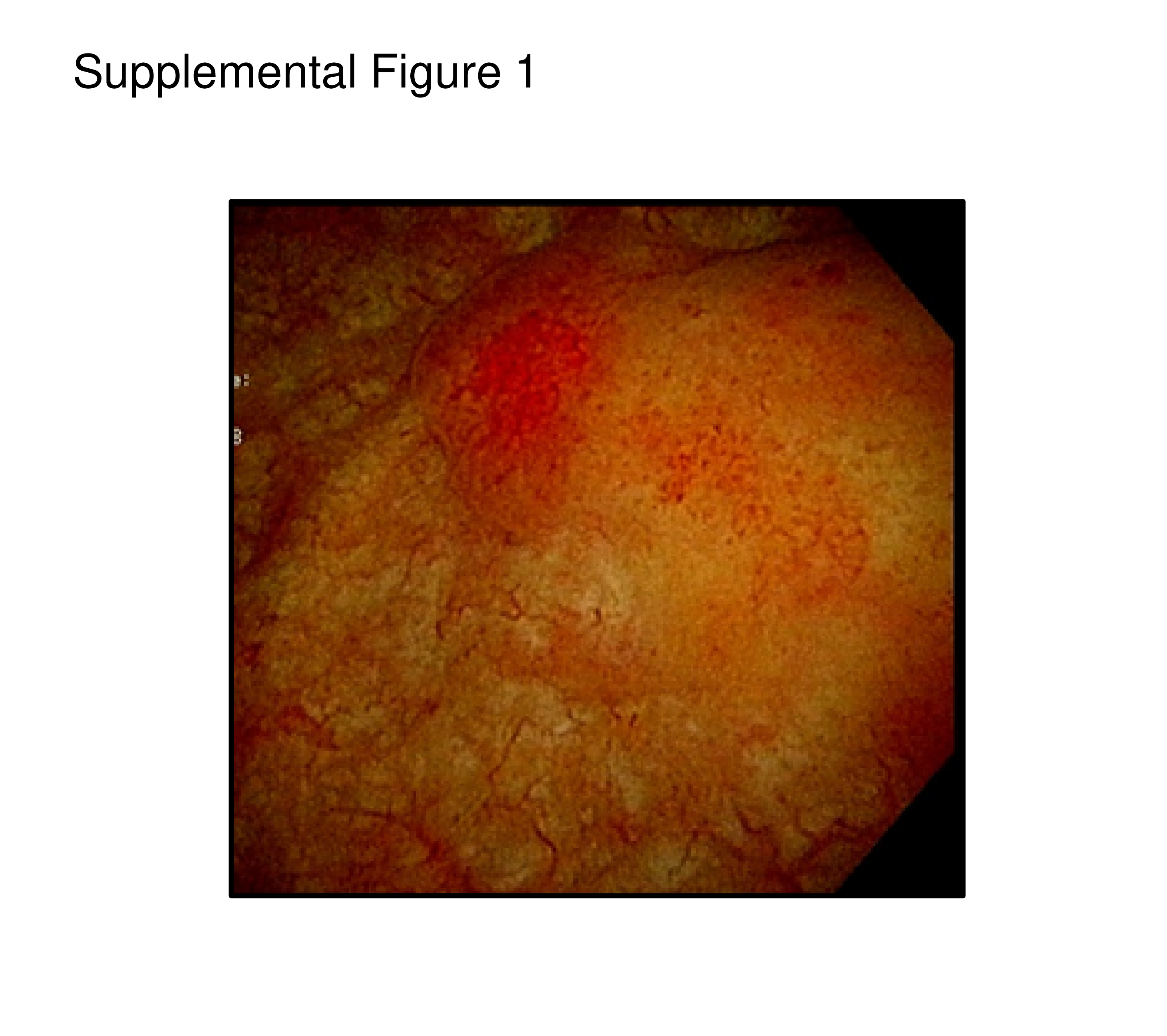

Supplement: Supplementary file 1 — Figure S1. Cystoscopic findings at the time of recurrence. Non‐papillary elevated lesions were found on the left wall of the bladder. [file IJU5-4-360-s002.tiff]

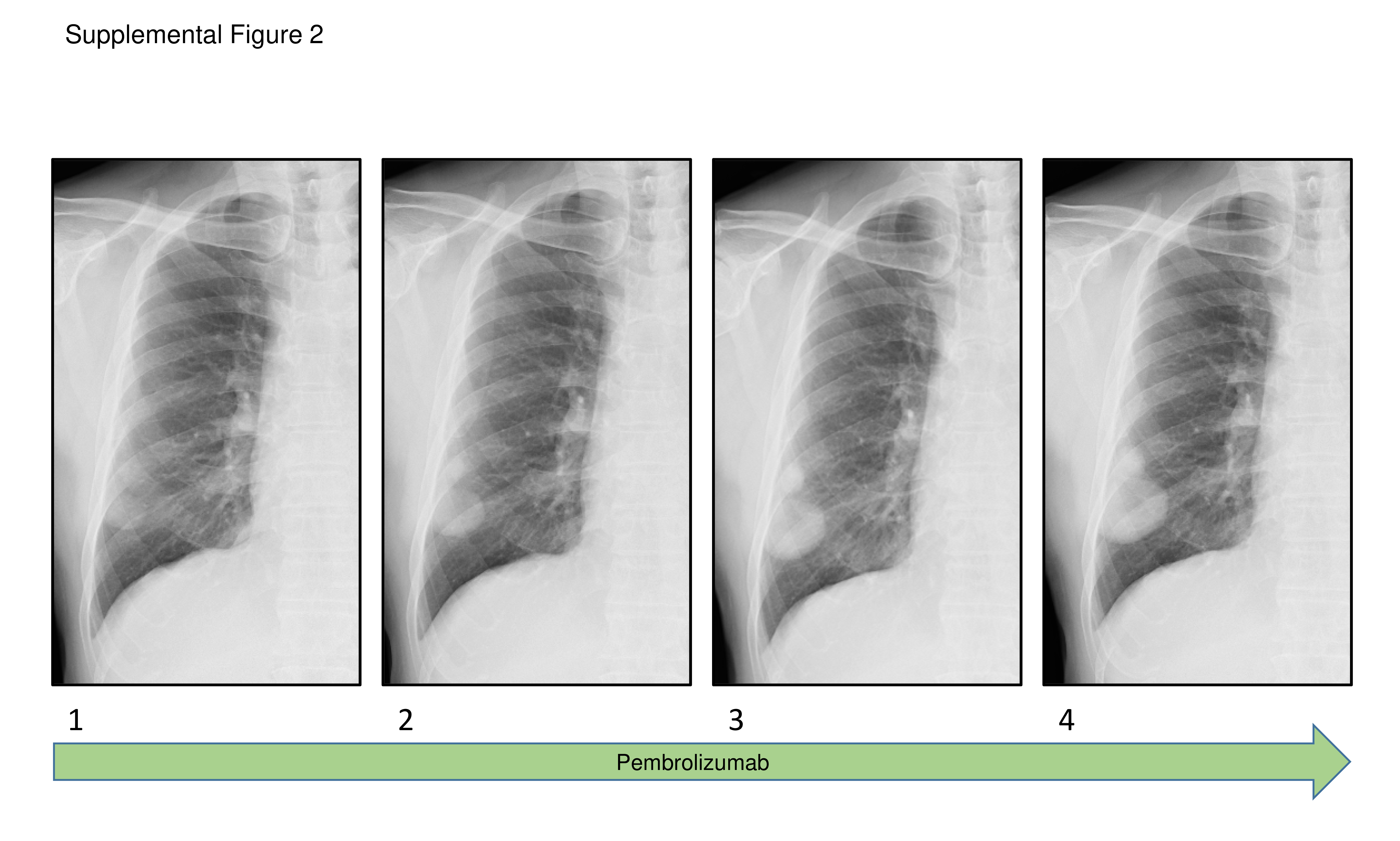

Supplement: Supplementary file 2 — Figure S2. Treatment course for lung metastases during pembrolizumab. Chest X‐rays taken each time during pembrolizumab showed a clear increase in lung metastases over time. [file IJU5-4-360-s001.tiff]
